# The role of semantic assessment in the differential diagnosis between late-life depression and Alzheimer’s disease or amnestic mild cognitive impairment: systematic review and meta-analysis

**DOI:** 10.1007/s10433-023-00780-z

**Published:** 2023-08-10

**Authors:** Sandra Invernizzi, Alice Bodart, Laurent Lefebvre, Isabelle Simoes Loureiro

**Affiliations:** 1 Departement of Cognitive Psychology and Neuropsychology, University of Mons, Mons, Belgium; 2 Fonds National de La Recherche Scientifique, Brussel, Belgium

**Keywords:** A systematic review, Semantic memory, Neuropsychology, Late-life depression, Alzheimer’s differential diagnostic, Fluency task, Meta-analysis

## Abstract

**Object:**

The cognitive complaints encountered in late-life depression (LLD) make it difficult to distinguish from amnestic mild cognitive impairment (aMCI) and Alzheimer's disease (AD) based on an analysis of neurocognitive disorders. The hypothesis of the early impairment of semantic memory in AD and aMCI is considered a potential differential cognitive clue, but the absence of this impairment has not yet been confirmed in  LLD.

**Method:**

Based on the PRISMA method, we systematically seek neuropsychological assessments of individuals with LLD, the present study included 31 studies representing 3291 controls and 2820 people with LLD. Wherever possible, studies that tested simultaneously groups with LLD, AD (or aMCI) were also included. The results of the group of neuropsychological tasks relying on semantic memory were analyzed in two groups of tasks with high- or low-executive demand. The mean average effect of LLD was calculated and compared to the incremental effect of aMCI or AD on the scores. Linear regressions including education, age, and severity and type of depression were run to seek their power of prediction for the mean average effects.

**Results:**

LLD has a medium effect on scores at semantic and phonemic fluency and naming and a small average effect on the low-executive demand tasks. Differences in education is a predictor of the effect of LLD on phonemic fluency and naming but not on semantic fluency or on low-executive demand tasks. Except for semantic fluency, aMCI did not demonstrate an incremental effect on the scores compared to LLD, while AD did, for all the tasks except phonemic fluency.

**Conclusion:**

Assessment of semantic memory can be a discriminating clue for the distinction between depression and Alzheimer’s disease but some methodological variables are highly influential to the scores, especially education. However, high-executive semantic tasks alone do not allow us to clearly distinguish LLD from AD or aMCI, as both pathologies seem to have a largely dialectical influential relationship, but low-executive semantic tasks appear as more sensible to this pathological distinction.

## Introduction

It is now largely recognized that the cognitive profile of late-life depression (LLD) is affected in a manner that resembles what can be encountered in Alzheimer’s disease (AD) and mild cognitive impairment (MCI).

AD is a major neurocognitive disorder encountering the criteria for a dementia syndrome listed in the Diagnostic and Statistical Manual of Mental Disorders, Fifth Edition [DSM-V] and affecting the activities of daily life (Petersen 2016; Srinivasan 2014). When affected by the early stage of AD, the patient’s most common results at the neuropsychological assessment demonstrate an impairment of episodic memory, language, attention, executive functions, and visuospatial abilities (Weintraub et al. 2012).

Furthermore, the prodromal phase of AD is often associated with MCI, which is determined by cognitive decline objectivated by normed tests, but for which the scores do not meet the criteria for dementia syndrome (Petersen et al. 1999). Not considered a neurodegenerative condition per se, MCI is itself a tricky condition that may or may not evolve into a neurodegenerative disorder (De Rotrou et al. 2005). Subdivided into four subtypes depending on the number of cognitive domains affected, MCI can exist as single-domain amnestic MCI (aMCI), multidomain amnestic MCI, single-domain non-amnestic MCI, and multidomain non-amnestic MCI (Díaz-Mardomingo et al. 2017). The focus of this paper is on the form of MCI that is currently identified as the most likely to progress to AD, namely aMCI, although the debate on the link between certain subtypes of MCI and their progression to one or another major neurocognitive disorder is still largely ongoing. Formerly identified as MCI of Alzheimer-type (Dubois and Albert 2004), also known as the hippocampal type, aMCI is essentially defined by an assessment of episodic memory with poor results at the free recall task, and an insufficient effect of cuing to help this recall despite adequate encoding.

In LLD, the extended damage to gray matter volume in the prefrontal, medial-temporal (Ballmaier et al. 2004; Kohler et al. 2010; Lamar et al. 2012), and subcortical cortices (Kohler et al. 2010; Lamar et al. 2012), results in cognitive impairment that also affects episodic memory (Elderkin-Thompson et al. 2006; Lamar et al. 2012) and executive functioning (Alexopoulos et al. 2005; Elderkin-Thompson et al. 2006; Henry and Crawford 2005; Morimoto et al. 2011; Rajtar-Zembaty et al. 2017; Snyder 2013), and, in a more secondary manner, attention (Lee et al. 2012; Rajtar-Zembaty et al. 2017), information processing (Lamar et al. 2012; Lee et al. 2012), learning (Lee et al. 2012), visuospatial (Lamar et al. 2012) and language (Rajtar-Zembaty et al. 2017) skills.

Consequently, these three pathological situations show important overlaps regarding discernible cognitive impairment. The diagnostical confusion is also increased by the fact that both aMCI and early stage of AD are frequently accompanied by important depressive symptoms that can influence the path of the disorder evolution, and by the fact that cases of LLD are sometimes considered as part of the prodromal phase of AD (for review see (Invernizzi et al. 2021). The role of depression in the evolution from MCI to AD is an object of interesting research, especially about the key role of loneliness (see Lara et al. 2019; Rozzini et al. 2008).

A possible improvement to the differential diagnosis would be the assessment of semantic cognition, according to its very specific impairment in AD and aMCI (Brambati et al. 2009, 2012; Brunet et al. 2011; Callahan et al. 2015; Joubert et al. 2020; Simoes Loureiro and Lefebvre 2016a, b).

Semantic cognition enables the retrieval and use of our general knowledge of the world by combing activation in the semantic representation system (Binder et al. 2009; Hoffman 2018; Jefferies et al. 2020), an executive processes allowing the controlled retrieval of less salient semantic information (Hoffman 2018) and the selection or inhibition of task-relevant aspects of semantic knowledge (Chiou et al. 2018; Hoffman 2018; Joyal et al. 2020). The activation of the semantic representation system is an automatic process almost sufficient for ongoing tasks requiring dominant or highly accessible semantic information (Chiou et al. 2018; Jefferies et al. 2020; Teige et al. 2018). However, the executive contribution to semantic retrieval includes more controlled processes that are increasingly needed when less easily accessed aspects of knowledge are required (Chiou et al. 2018; Jefferies et al. 2020; Teige et al. 2018).

As these elements of semantic cognition rely on distinct but interacting brain regions (Copland et al. 2007; Joyal et al. 2020; Raucher-Chene et al. 2018, 2017), the full processing of semantic cognition can be almost equally impaired by a broad number of neuropathological conditions, while the separate subprocesses should be more specifically impaired by some conditions and not others.

First, the activation of the semantic representation system (Binder et al. 2009; Hoffman 2018; Jefferies et al. 2020) is expected as being affected only by AD (or aMCI). Indeed, this activation relies on the anterior temporal cortices (Binder and Desai 2011; Binder et al. 2009; Jefferies et al. 2020; Sami et al. 2018; Venneri et al. 2008) and parahippocampal regions damaged by the neuropathological injuries of AD (Venneri et al. 2008). For a review of neuroanatomical correlates of semantic impairment in AD, refers to Venneri et al. (2008).

Secondly, the executive processes allowing semantic retrieval are associated with a network comprising the inferior prefrontal cortex, posterior middle temporal gyrus, and interparietal sulcus (Binder et al. 2009; Hoffman 2018; Venneri et al. 2008). Many psychopathological or neuropathological diseases including LLD and AD can damage one of these brain regions. Consequently to the neural differentiation, when it comes to the impairment of these subprocesses by LLD and AD, one must expect that both activation of the semantic representation system (concepts and links) (Laisney et al. 2009, 2011; Simoes Loureiro and Lefebvre 2016a, b) and executive processes of semantic retrieval will be impaired in AD, while only executive processes will be touched by LLD. Indeed, the regions involved in the activation of the semantic representation system, namely, the anterior temporal cortices (Binder and Desai 2011; Binder et al. 2009; Jefferies et al. 2020; Sami et al. 2018; Venneri et al. 2008) and parahippocampal regions, have not been reported to be affected by the neuropathological consequences of LLD. This leads to the expectation that if LLD affects semantic retrieval competencies it is most likely related to the dysexecutive consequences of the loss of volume of the superior frontal gyrus and ventromedial frontal cortex (Alexopoulos et al. 2005; Boccia et al. 2015; Morimoto et al. 2011). It is admitted that LLD affects the domain-general executive selection system involved in inhibition tasks, such as the Stroop test (Hoffman 2018), and that impaired results in recall tasks in LLD rely on impairment of the semantic executive (clustering) implied in the encoding phase of the task (Elderkin-Thompson et al. 2006; Lamar et al. 2012).

This opportunity to discriminate the semantic impairment due to AD or LLD then requires investigating in which manner these two pathological situations' cognitive impairments are reflected in the performance of semantic-based neuropsychological tasks. Following the above reasoning, it becomes relevant to consider the use of these neuropsychological tests in medical practice. To do so, let us consider two different types of tests that involve mainly, even if among other cognitive resources, semantic knowledge while attempting to assume that certain tasks can be completed with minimal contribution from executive semantics, while others are highly dependent on it.

In this systematic review of literature, we will consider that semantic cognition can therefore be assessed with two main groups of neuropsychological tests, which require more or less one of both semantic retrieval subprocesses: activation in the semantic representation system (Binder et al. 2009; Hoffman 2018; Jefferies et al. 2020), and executive processes.

The first group of tasks involves both the activation of the semantic representation system and an important contribution of executive processes. It includes mainly confrontation naming and verbal fluency tasks. Confrontation naming (e.g., Boston Naming Test (Kaplan et al. 2001), DO 80, or LEXIS denomination subtest (de Partz de Courtray et al. 2001)) is a task requiring the name of a given image and assessing the capacity to retrieve the meaning of a concept and its lexical label. Recognition via the activation of the semantic representation system also requires an executive contribution to actively retrieve the lexical label for the verbal production of the answer (Higby et al. 2019). However, in neuroimaging research seeking the correlations between brain region disruptions in AD and impaired results in confrontation naming, Domoto-Reilly et al. (2012) demonstrated that, even if a general relationship could be demonstrated with a region of interest distributed between the frontal, parietal, and temporal cortices, thinning of the left anterior temporal lobe (directly linked to semantic representation system) was more highly correlated with impaired naming performance (Domoto-Reilly et al. 2012).

The verbal fluency task, which is almost always included in general cognitive assessments, is auto-initiated, sometimes classified as a semantic measure or a language measure, or a measure of executive functioning. In fact verbal fluency is all of these, because to perform it (to produce the maximum of words according to a given rule), either phonemic (all words have to start with the same letter) or semantic (all words have to belong to a given category), one needs to activate the lexicosemantic network (by activation of the semantic representation system), but also to maintain and refresh information in the working memory with an important contribution from the executive functions (Henry and Crawford 2005, 2004). The participant has to self-initiate the retrieval, keep track of the responses already given, and inhibit responses that are inappropriate and switch to another group of concepts when the first one is exhausted (e.g., in semantic fluency “animals,” switching from house pets to farm animals). Despite this multi-determined aspect of fluency, at bases, it is first of all impossible to perform without functional access to the semantic representation system. It has been extensively studied in healthy elderly individuals and patients with AD through various meta-analyses (Henry and Crawford 2004; Laws et al. 2007, 2010). Regarding the healthy elderly, it is assumed that they have better verbal fluency performance in the semantic task than in the phonemic task, and that this advantage persists over time (Chasles et al. 2019; Vaughan et al. 2016). Regarding AD, two meta-analyses (Henry and Crawford 2005, Laws et al. 2007) showed that the effect for both AD (Laws et al. 2007) and LLD (Henry and Crawford 2005), the deleterious effect on semantic fluency was significantly larger than on phonemic fluency (and confrontation naming) which is the inverse result of healthy aging participants. Notice that the samples in Henry and Crawford’s research were composed of adults and not the elderly (age: 52.7 ± 13.69) (Henry and Crawford 2005) and that the authors have shown that the contradictory results are essentially the consequence of methodological artifacts (highly influenced by intra-individual variables such as education or lexical background) and may partly reflect executive dysfunction, but also be a consequence of more generalized cognitive impairment (Henry and Crawford 2005). The effect of LLD on the results of verbal fluency and confrontation naming is however still unclear (Balsamo et al. 2018; Henry and Crawford 2005; Lee et al. 2012).

The second group includes tasks that allow to reduce of as much as possible cognitive (and especially executive) contributions, other than activation of the semantic system. It regroups the tasks that are not auto-initiated. In this category are semantic pairing and classification, designation and recognition tasks, lexical decision tasks, semantic knowledge, and vocabulary descriptions. Semantic pairing tasks require pairing images based on their semantic relationships (e.g., Pyramid and Palm Tree Test (Klein and Buchanan 2009), Camel and Cactus Test (Adlam et al. 2010)) while in classification the participant must classify images among superordinated categories (e.g., classification in Cambridge Semantic Memory Test Battery (Adlam et al. 2010) or LEXIS subtest (de Partz de Courtray et al. 2001), Size Weight Attribute Test (Warrington and Crutch 2007)). A particular type of recognition task is the lexical decision tasks (e.g., Spot-the-word (Baddeley et al. 1993)) in which participants see pairs of items comprising one word and one non-word and must identify the word. Yuspeh and Vanderploeg (2000) suggested that spot-the-word is a useful additional measure to estimate premorbid intelligence (Yuspeh and Vanderploeg 2000). When used as a semantic assessment, this usefulness can be argued against by the fact that in AD, patients are able to discriminate a word from a non-word, even if the meaning of the word is lost.

Semantic knowledge questionnaires consist of multiple choices about concepts, either about their subordinated category or their features (e.g., the Semantic Knowledge Questionnaire (Simoes Loureiro and Lefebvre 2015)) and can also contain questions about famous persons or famous public events. Last of this group are the vocabulary (e.g., Mill Hill Vocabulary Scale (Colman 2009)) and similitude tasks, often part of the verbal subtests in general intelligence scales (e.g., Wechsler Adult Intelligence Scale (Hartman 2009)). Note that in the literature, including research presenting a general cognitive assessment, some studies present the same tests exposed in this paragraph, not as part of a semantic, but as part of a language variable (Butters et al. 2004; Hoffman 2018; Yuspeh and Vanderploeg 2000). The responses for the tasks of this first group are demonstrated as being affected by the early stages of AD (Adlam et al. 2010; Croisile et al. 2011; Hernández et al. 2008; Perri et al. 2019, 2012; Zannino et al. 2015) and MCI (Belanger and Belleville 2009; Joubert et al. 2020; McLaughlin et al. 2014) while the issue of their impairment in LLD is rarely examined.

Given this information, several questions arise. Firstly, is it possible to detect an effect of LLD on the scores on the semantic evaluation tests? Secondly, is it possible to discriminate between an effect of LLD on the tests that are included in the first and second groups? Finally, when studies compare LLD to AD (or aMCI), will the effect on semantic test scores be significantly different between the two conditions? To answer the first question, we systematically searched studies enabling us to identify outcomes at the semantic assessments of groups of patients with LLD, compared to elderly adults without cognitive impairment. To answer the second question, the results of these tests have been analyzed comparatively according to their belonging to the first or second group. To answer the last question, we also searched the literature for articles that compared the results of groups of LLD to those of groups of AD (or aMCI) within the same research, in order to see if the effect of AD (or aMCI) on these results was significantly greater than that of LLD.

## Method

This systematic review was conducted following the guidelines of the Preferred Reporting Items for Systematic Reviews and Meta-Analyses (PRISMA) (Liberati et al. 2009) and the guidelines for the contents of a Cochrane diagnostic test accuracy protocol (Deeks et al. 2013). The final protocol of the systematic review was submitted for registration at the PROSPERO International Prospective Register of Systematic Reviews on July 13, 2021 (register code CRD42021266476). The management of methodological steps was performed using Excel and EndNote. Intermediate tables are provided upon request.

We applied two different research strategies. The first search strategy was designed to find research enabling to identification outcomes at the semantic assessments of groups of patients with LLD, compared to elderly adults without cognitive impairment or depressive symptoms. The research identified with this strategy were included to answer the two first questions; is it possible to detect an effect of LLD on the scores on the semantic evaluation tests, and is it possible to discriminate between an effect of LLD on the tests that are included in the first and second groups? The second research strategy allowed us to identify studies that compare LLD to AD (or aMCI) to see if the effect on semantic test scores is significantly different between the two conditions.

The accepted format was non-interventional psychophysiological research, written in English or French, including case–control and cross-sectional studies. Longitudinal or retrospective studies were also included if they allowed for the extraction of a neuropsychological measurement of the groups when they were over 60 years old. To be included, the research must provide at least one measure of semantic or semantic-executive tests for at least two groups: LLD and control, LLD and AD, or LLD and MCI. Note that the presence of data regarding LLD was the main inclusion criterion. Research with results of AD (or aMCI) were included only if they allowed a direct comparison with a group with LLD, otherwise they were not included (and will be the object of a different systematic review by the same research team). Were excluded or the review articles that (1) did not use a semantical assessment or used a semantical assessment not validated by a previous publication, (2) allowed only the extraction of data for a population under 60 years old, (3) did not include a possibility of relating semantical data with a LLD group, (4) included only an AD or aMCI population without an LLD group, (5) included only a group of LLD without a control group, (6) were meta-analyses or systematic reviews.

**Fig. 1:**
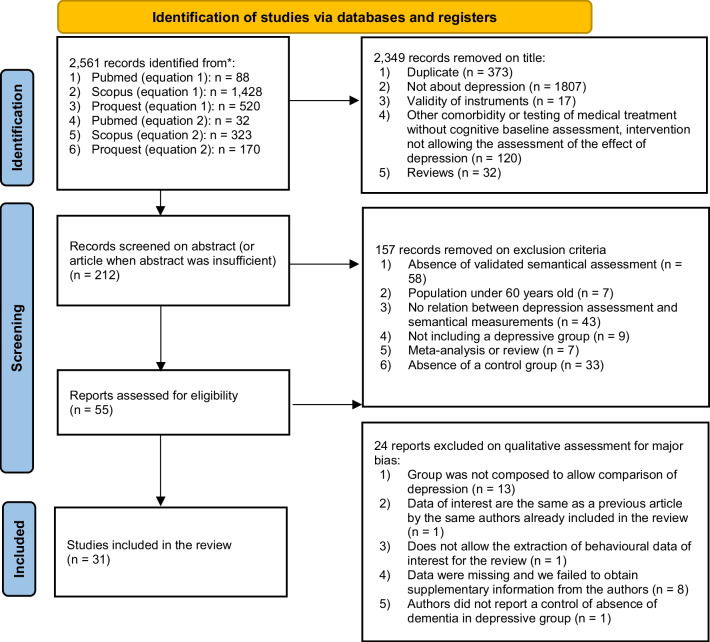
PRISMA Flow diagram of study screening and selection. *Note.* The first round of selection was performed at the beginning of the systematic review process in August 2021 with no limitation in years of publication. A second round of research was performed at the end of the systematic review process in June 2022 with a limitation on articles published in 2021 or 2022

As reported in the flow chart (Fig. 1), at the end of the selection procedure, 55 articles were selected by two independent researchers who evaluated the quality of the selected research based on the criteria decided in the research protocol registered on PROSPERO. The quality assessment frame was created by the research team, and quality criteria were determined to identify the risks of selection and diagnostic bias, the possible presence of confounders for data, and the accuracy of data collection and statistical analysis. Studies were excluded from the review because they did not meet the criteria for individual selection or were not representative of the target population. Twenty-four studies were removed because they did not meet one of the quality criteria (Fig. 1), and a systematic review of the content was conducted on 31 articles.

### Data extraction and analysis

R scripts and detailed databases are accessible on Open Science Framework (OSF): https://osf.io/evrwf/?view_only=935fb2bcf5aa487f90ec22a034835b0a

We systematically extracted from the 31 articles (presented in Table 1) the number of participants of each group and, in means and standard deviations, the ages, degree of depressive symptoms, quality of depression, level of education and information about the diagnosis of depression. When the studies included only groups with LLD (and no groups of AD and aMCI), we reported the information about the control of non-dementia. When the studies also included groups with aMCI or AD, we reported information about the quality of these diagnosis. The complete table of data extracted from the articles are available on OSF. From these 31 studies we extracted 52 groups comparisons; 31 comparisons between LLD and control groups, 7 comparisons between aMCI and LLD groups, and 4 comparisons between AD and LLD groups. Were compared results at the tests of semantic (*n* = 50) and phonemic fluency (*n* = 36), and confrontation naming (*n* = 30), corresponding to the first group of tasks that involves both the activation of the semantic representation system and an important contribution of executive processes. For the second group of tasks that rely more directly to the activation of the semantic system, we extracted the subtest of the language of the ACE-R (Mioshi et al. 2006) (*n* = 3), the Pyramid and Palm Tree Test (Klein and Buchanan 2009) (*n* = 9), the spot-the-word (Yuspeh and Vanderploeg 2000) (*n* = 3), the similarities subtest of the *Wechsler Adult Intelligence Scale (*WAIS) ^[65]^ (*n* = 2), vocabulary subtest of the Wechsler Abbreviated Scale of Intelligence (WASI) (McCrimmon and Smith 2013) (*n* = 3), and the vocabulary subtest of the WAIS (Hartman 2009) (*n* = 1). All the tests of the second group were aggregated together in the same analysis of the gathered effect.

**Table 1:** Thirty-one review articles with first author, year of publication, group characteristics, type of results reported, reference for the diagnostic of depression, and scale of depressive symptoms

First author, year	Groups	Diagnostic criteria LLD	Dep. scale	Extracted tests	RB1	RB2	RB3
Ravdin et al. (2003)	LLD moderate	Symptoms	GDS 30	Semantic fluency; phonemic fluency	*	*	
Butters (2004)	LLD	DSM IV	SCID	Semantic fluency; phonemic fluency; naming; spot-the-word			
Elderkin-Thompson (2006)	LLD	DSM IV	HDRS	Semantic clustering in recall			*
Mah (2017)	LLD	DSM V	GDS 15	Semantic fluency; phonemic fluency; naming; Wais vocabulary			
Fischer (2008)	LLD	DSM IV	GDS 30	Semantic fluency; phonemic fluency; similarities; Wais similitudes			
Avila (2009)	LLD (high—low education)	DSM IV	HDRS	Semantic fluency; phonemic fluency			
Dillon (2009)	LLD eary onset						
	LLD late onset	DSM IV	HDRS	Semantic fluency; phonemic fluency; naming			
Barabassy (2010)	LLD	DSM IV	MADRS	Semantic fluency; naming			*
Parra et al. (2010)	LLD						
	AD	DSM IV	GDS 30	Semantic fluency; phonemic fluency	*	*	
Brunet (2011)	LLD						
	aMCI						
	aMCI D +	DSM IV	GDS 30	Semantic fluency; phonemic fluency; naming; PPTT			
Dillon (2011)	Dysthymia						
	Subsynd. LLD						
	LLD due to AD	DSM IV	HDRS	Semantic fluency; phonemic fluency; naming			
Elderkin-Thompson (2011)	LLD	DSM IV	HDRS	Semantic fluency; phonemic fluency			
Jungwirth (2011)	LLD	DSM IV	HDRS 17	Semantic fluency			
Alves (2012)	LLD	DSM IV	GDS 15	Semantic fluency; naming			
Lim et al. (2012)	LLD	DSM-IV TR	HDRS 17	Semantic fluency; naming			
Tam and Lam (2012)	LLD						
	MCI	DSM IV	HDRS 24	Semantic fluency			*
Callahan (2015)	LLD						
	aMCI						
	aMCI D +	DSM IV	GDS 30	Semantic fluency; phonemic fluency; naming; PPTT			
Koenig (2015)	Euthymic	DSM IV	HDRS 17	Semantic fluency; phonemic fluency; naming; spot-the-word			
Rotomski (2015)	LLD						
	AD	ICD 10	not referred	Semantic fluency phonemic fluency subtest language ACE R	*	*	
Beckert (2016)	LLD	DSM IV	GDS 15	Semantic fluency; phonemic fluency; naming; subtest language ACE R			*
Callahan (2016)	LLD						
	aMCI						
	aMCI D +	DSM IV-R	GDS 30	Semantic fluency; phonemic fluency; naming; PPTT			
da Costa Dias (2017)	LLD	DSM IV	GDS 15	Semantic fluency; naming			
Rajtar-Zembati (2017)	LLD	DSM V	GDS 15	Semantic fluency; phonemic fluency			*
Esteves (2019)	LLD	Symptoms	GDS 15	Semantic fluency; phonemic fluency; naming	*		*
Morin et al. (2019)	LLD	DSM IV	HDRS 24	Semantic fluency; phonemic fluency; naming; Wais similarities			
Olaya et al. (2019)	LLD lifetime						
	LLD remittent						
	LLD incident						
	LLD persistent	DSM IV	SCID	Semantic fluency	*	*	*
Lin et al. (2020)	LLD	DSM V	GDS 15	Semantic fluency			
Muniswamy et al. (2020)	LLD	DSM IV R	GDS 30	Semantic fluency			*
Faoro (2021)	LLD	Symptoms	GDS 30	Semantic fluency; naming; wais vocabulary	*		
Ramos-Henderson et al. (2021)	LLD	Symptoms	GDS 15	Semantic fluency; phonemic fluency	*	*	
Liu et al. (2022)	LLD early onset						
	LLD late onset	DSM IV	GDS 15	Phonemic fluency; naming			

*Note*. Groups: identification of the pathological groups for which data were extracted in the meta-analysis. Diagnostic criteria LLD: referred criteria of diagnostic for Major Depression; Diagnostic and Statistical Manual of Mental Disorders (DMS), version 4 (IV), 4 revised (IV R) and 5 (V); International Statistical Classification of Diseases and Related Health Problems, 10th Revision (ICD 10), or not stated, but only referred to according to the threshold of significance of the depressive scale (Symptoms). Dep. scale represents the depression scales reported by the authors to measure the level of depressive symptoms, including the geriatric depression scale (GDS), the Hamilton depression rating scale (HDRS), the Montgomery-Åsberg Depression Rating Scale (MADRS) and the Structured Clinical Interview for DSM Disorders (SCID). Risks of bias are indications of the fact that researches do not entirely respect the non-exclusion criteria of quality of the research; (1) do not state the precise condition of LLD diagnostic of the level of depressive symptoms of the pathological group; (2) state control of non-dementia but do not provide the precise criteria of control; (3) data about the results of the control group were missing and managed by statistical procedure

### Statistical analysis

Meta-analysis of the results was conducted using the metafor package (Viechtbauer 2010) with a random effects model using the rma function. The preferred choice for the random effects meta-analytic model was decided due to the important differences in sample sizes between studies and between groups within studies, which allowed us to assume random variation in the effect of interests among studies. Moreover, Viechtbauere (2010) recommended the choice of a random effects model to correct a plausible selection bias, which is the consequence of the absence of unpublished studies in our selection of research (Viechtbauer 2010). Unlike the fixed-effects model, which provides an inference only suitable for the sample of selected studies, the random effects model provides an inference about the average effect in the entire population of studies from which the included studies are assumed to be a random selection (Viechtbauer 2010).

The observed measure is the *weighted average effect,* expressed with the Hedge’s g (Hedges 1981) and its derived prediction interval* (95% IC)*. This result is reported with the *p-*value of significance calculated using a Z score. The 95% prediction interval provides the range in which the point estimate of 95% of future studies will fall, assuming that true effect sizes are normally distributed throughout the domain (Borenstein et al. 2009). The heterogeneity between studies included in one calculation of the combined effect was reported using the *I*^2^ value of heterogeneity (Higgins and Thompson 2002). This value reports the amount of variability that cannot be justified by the sampling differences between the studies. According to the rule of thumb, the heterogeneity is considered as low (*I*^2^ = 25%), moderate (*I*^2^ = 50%) or substantial (*I*^2^ = 75%).

Since sampling variation is important between and within studies, the combined effect size was calculated with the SMDH function for the *standardized mean difference* with heteroscedastic population variances in the two groups (Bonett 2009). The effect sizes are reported in Tables 2, 3, 4 and 5. For the fluency and naming tasks, a single effect size on each test was calculated. For the tests of group two, given their small number, a pooled effect size was measured for all the tests. In Table 2 and 3 the effects of LLD compared to a control group are reported, and in Tables 4 and 5 the differences in effects between the depressed groups and the groups with either aMCI or AD are reported. As explained above, this research did not include direct comparisons between aMCI or AD and control groups, as the aim of this research was either to measure the effect of LLD or to identify a direct comparison between the conditions (LLD vs aMCI/AD).

**Table 2:** Standardized mean difference between Control and LLD groups on the results of the tasks

41 studies including LLD and control		
	Control	LLD
N	5972	2561
Age	69.57 (6.52)	69.48 (7.06)
Education	11.46 (3.48)	10.53 (3.85)
Dep. Sympt	2.67 (2.37)	13.26 (4.8)

			Degree of freedom	Effect size Hedge's g (95% CI)	p-value	Heterogeneity (*I*^2^)
Phon. fluency (high-exe.)	27.44 (6.9)	24.55 (7.28)	23	0.46 (0.28 to 0.63)	0.001	81.03%
Sem. fluency ﻿(high-exe.)	22.34 (5.5)	20.03 (5.84)	34	0.46 (0.32 to 0.57)	0.001	79.90%
Naming ﻿(high-exe.)	36.74 (4.25)	32.92 (6.46)	21	0.54 (0.36 to 0.73)	0.001	79.69%
PPTT (low-exe.)	42.77 (1.91)	42.77 (2.84)	2	0.23 (**−**0.17 to 0.63)	ns	0%
Spot-the-word (low-exe.)	50.37 (7.66)	47.46 (9.24)	3	0.13 (**−**0.08 to 0.42)	0.03	66.52%
Vocabulary (low-exe.)	46.43 (11.46)	41.87 (9.51)	3	0.44 (0.14 to 0.73)	ns	0%
Similarities (low-exe.)	16.35 (2.99)	16.84 (2.54)	1	0.24 (**−**1.13 to 0.64)	0.02	81.43%
ACE-R Language (low-exe.)	23.56 (2.17)	23.21 (2.73)	0	0.14 (**−**0.12 to 0.41	ns	0%
Semantic cluster (low-exe.)	1.70 (0.90)	1.25 (1.15)	1	0.45 (0.18 to 0.73)	ns	0%
pooled low-exe. tasks	39.91 (6.90)	37.88 (6.70)	13	0.20 (0.05 to 0.34)	0.01	44.32%

**Table 3:** Linear regression models for prediction of the average effect of LLD on the tasks

Average effect of LLD on:	Fitted regression model	Significance of overall regression
Semantic fluency	= 0.48 + 0.65*yEdu—0.006*yDS + 2.54*yAge	*R*^*2*^ = 0.02, *F(*3.42) = 0.35,* p* = 0.78 (ns)
Phonemic fluency	= 0.32 + 1.66*yEdu—0.84*yDS + 0.24*yAge	*R*^*2*^ = 0.27. *F(*3.28) = 3.37,* p* = 0.03
Naming	= 0.48 + 1.80*yEdu + 0.02*yDS – 6.90*yAge	*R*^*2*^ = 0.33, *F(*3.26) = 4.20,* p* = 0.01
Pooled low-executive tasks	= 0.21 + 0.46*yEdu—0.03*yDS – 5.32*yAge	*R*^*2*^ = 0.11, *F(*3.17) = 0.71,* p* = 0.55 (ns)

This table report the results of the multiple linear regression used to test if the difference between LLD and control groups regarding the level of education (yEdu), of depressive symptoms (yDS) and mean age (yAge) significantly predict the average effect of LLD on the tasks of semantic fluency, phonemic fluency, naming and the pooled effect of the low-executive tasks. The fitted models were only significant for phonemic fluency and naming. (ns) indicates a p-value non significant.

**Table 4:** Standardized mean difference between control and LLD groups, and between LLD and aMCI groups on the results of the tasks

7 studies including LLD and aMCI			
	Control	LLD	aMCI
N	375	73	142
Age	69.84 (6.21)	71.47 (9.36)	70.55 (7.07)
Education	12.16 (3.42)	11.31 (3.68)	11.24 (3.70)
Dep. Sympt	3.11 (2.76)	12.31 (4.81)	10.49 (5.37)

High-executive tasks:					Degree of freedom	Hedge's g (95% CI)	p-value	Heterogeneity (*I*^2^)
Phonemic Fluency	28.25 (8)	23.88 (9.03)	23.28 (9.28)	Ctrl//aMCI	6	0.52 (0.28 to 0.76)	0.001	0%
				Ctrl//LLD	6	0.42 (0.18 to 0.66)	0.02	0%
				LLD//aMCI	6	−0.01 (0.24 to −0.76)	ns	0%
Semantic Fluency	14.7 (5.79)	12.67 (6.53)	10.25 (3.16)	Ctrl//aMCI	6	0.97 (0.62 to 1.33)	0.001	51.14%
				Ctrl//LLD	6	0.39 (0.14 to 0.64)	0.001	0%
				LLD//aMCI	6	0.29 (0.04 to 0.55)	0.02	6.42%
Naming	16.06 (1.48)	14.29 (3.25)	15.09 (1.63)	Ctrl//aMCI	6	0.49 (0.26 to 0.73)	0.001	0%
				Ctrl//LLD	6	0.43 (0.19 to 0.67)	0.001	0%
				LLD//aMCI	6	0.04 (0.45 to **−**0.36)	ns	61.82%
Low-executive tasks:								
PPTT	49.88 (1.83)	48.87 (2.64)	47.88 (2.35)	Ctrl//aMCI	5	0.65 (0.10 to 1.21)	0.03	78.25%
				Ctrl//LLD	5	0.21 (−0.04 to 0.47)	ns	0%
				LLD//aMCI	5	−0.28 (−0.73 to 0.15)	ns	61.47%
Vocabulary	56.87 (6.01)	49.23 10.20)	53.73 (5.01)	Ctrl//aMCI	0	0.55 (−0.13 to 1.26)	ns	**−**
				Ctrl//LLD	0	0.89 (0.15 to 1.92)	0.01	
				LLD//aMCI	0	0.54 (−0.16 to 1.26)	ns	
Pooled low-exe. tasks	50.88 (2.24)	48.92 (3.72)	48.72 (2.73)	Ctrl//aMCI	6	0.64 (0.16 to 1.11)	0.008	73.57%
				Ctrl//LLD	6	0.28 (0.05 to 0.52)	0.02	0%
				LLD//aMCI	6	−0.17 (−0.79 to 0.26)	ns	65.51%

**Table 5:** Standardized mean difference between control and LLD groups, and between LLD and AD groups on the results of the tasks

4 studies including LLD and AD			
	Control	LLD	AD
N	287	264	292
Age	68.96 (6.54)	69.83 (7.85)	71.93 (6.89)
Education	10.57 (3.17)	9.60 (4.18)	11.15 (3.41)
Depr. Symptoms	1.90 (1.70)	17.18 (6.36)	16.60 (5.80)

High-executive tasks:					Degree of freedom	Hedge's g (95% C,I,)	p-value	Heterogeneity (*I*^2^)
Phonemic fluency	10.36 (2.52)	7.07 (3)	5.09 (2.46)	Ctrl//AD	2	1.76 (0.88 to 1.46)	0.001	0%
				Ctrl//LLD	2	1.23 (0.97 to 1.50)	0.001	0%
				LLD//AD	2	**−**0.002 (−0.27 to 0.27)	ns	10.32%
Semantic fluency	12.15 (3.65)	9.19 (2.65)	6.14 (2.47)	Ctrl//AD	2	1.65 (1.26 to 2.04)	0.001	24.91%
				Ctrl//LLD	2	0.55 (**−**0.55 to 1.65)	ns	92.05%
				LLD//AD	2	1.37 (0.89 to 1.85)	0.001	50.22.%
Naming	51.60 (4)	44.50 (7.20)	37.40 (10.10)	Ctrl//AD	0	1.81 (0.98 to 2.46)	0.001	/
				Ctrl//LLD	0	1.20 (0.62 to 1.78)	0.001	
				LLD//AD	0	0.79 (0.14 to 1.44)	0.01	
Low-executive tasks:								
ACE-R language	23.56 (2.17)	23.21 (2.73)	17.21 (4.04)	Ctrl//AD	0	1.94 (1.57 to 2.33)	0.001	/
				Ctrl//LLD	0	0.14 (**−**0.12 to 0.41)	ns	
				LLD//AD	0	1.73 (1.38 to 2.08)	0.001	

Due to the high level of heterogeneity in the comparisons between LLD and control groups, we used a model of linear regression to seek the influence of the side-variables age, level of depressive symptoms and level of education. To do so, we calculated three ratio indexes (yAge, yEdu and yDS) as the proportion between the value in the control group minus the value in the experimental group, divided by the value in control group. Analysis were run with the lmer (Kuznetsova et al. 2015) and the afex (Singmann et al. 2015) packages. We introduced in the model the medium effect size of depression on the results at the tests (yi calculated with the SMDH function) as the dependent variable; the three ratio index (yAge, yEdu and yDS) as fixed independent variables, and the specificity of LLD group (e.g., Major, minor, late onset, persistent, remittent, insistent, subsyndromal, dysthymia) as random effects. First, the structure of the mixed-effect modelling was determined by structuring a full model that included the fixed effects of interest and the random effects. The full model was then challenged with an ANOVA-like table for tests of random effect terms in the model and simplified until all the remaining intercepts and slopes of random effects were considered as significantly useful for the model.

## Results

### Effect of LLD on high- and low-executive semantic tasks

The results in Table 2 show the pooled effects of depression compared to control participants of the same average age and education level. We see that scores on the fluency and naming tasks are significantly affected by depression, but in each case with substantial heterogeneity between studies, which cannot be due to differences in sample sizes within each study.

We see in the same table that the tasks in low-executive group are also affected by depression, but with a smaller effect size, however, related to studies with less heterogeneity (*I*^2^ = 44.32%). The detail of this pooled result for the low-executive tasks should, however, be considered with caution, as it is dependent on very small numbers of research. When examined in detail, we see that several of these low-executive tasks are not significantly affected by depression, such as the PPTT, the vocabulary of the intelligence scales, the language sub-score of the ACE-R and the semantic cluster in a free recall task.

To investigate the possible sources of heterogeneity across studies, we performed linear regressions in which the dependent variable was the effect size of depression on the fluency, semantic and phonemic, naming and low-executive tasks, and the independent variables were the ratios calculated to represent the magnitude of the differences in age, education and depression level between the groups. The larger these ratios are, the greater the difference in the target data (e.g., education level) between the LLD group and the control group within the same study. We also ran these regressions in mixed fixed-effect, random effect models, where the random effect was the specific type of depression reported in each study (late onset, major, minor, subsyndromal, persistent, incident, remittent, lifetime, dysthymic, euthymic). For each mixed linear regression (semantic fluency, phonemic fluency, naming, low-executive), a maximum likelihood ratio analysis showed that the model including this random effect of the specific type of depression did not better explain the variance of the effect sizes than a model including only the fixed effects of the ratios age, education level and level of depressive symptoms. Therefore, the presented results are the outcomes of the linear regressions including only the fixed effects.

The outcome of these linear regressions, presented in Table 3, shows that the models are only significant in predicting the effects of LLD on phonemic fluency and naming tasks. When we observe the levels on these linear regression, we can see that the variable yEdu (difference in education level between the LLD group and the control group) significantly predicts the effects on the phonemic fluency task (*β* = 1.66, *p* = 0.02) and the naming task (*β* = 1.80, *p* = 0.04), while the variable yAge significantly predict the effects on the naming task *(β* = − 6.90, *p* = 0.02). Ratios related to level of depressive symptoms did not predict these results.

### Incremental effect of aMCI and AD on high- and low-executive tasks

Tables 4 and 5 show the results of the averaged effects when, in the same research, LLD groups were compared with both control and pathological groups suffering from either aMCI (Table 4) or AD (Table 5). The outcomes of Table 4 show that aMCI has no additional effect on the scores of the different tasks compared to the effect of LLD on these tasks. The only notable exception is semantic fluency, for which we see a significant medium-sized effect of the aMCI compared to the LLD. We interpret these results as showing that, apart from semantic fluency, none of the listed tasks show a different effect of the aMCI on individuals' performance or allow them to be distinguished from LLD results.

Table 5 shows that, except the phonemic fluency task, all the tasks considered show a significant effect of AD on the results when compared to LLD results. Our interpretation of this result is that phonemic fluency is not a task that shows a significant difference in results between LLD and AD, but that the semantic fluency, naming and language subtest of the ACE-R do.

### Comparison of results on high-executive tasks and methodological differences in studies

The aggregate means show that scores at phonemic, semantic fluency and naming are affected by the LLD status. However, for semantic fluency, if we compare aMCI groups to LLD groups, the aMCI has a significant incremental effect on the impairment of outcomes. This additive effect of the aMCI on LLD scores is not true for the phonemic fluency and naming tasks. This means that when the groups are tested with these three tasks, semantic fluency will show a marked difference between aMCI and LLD scores, while phonemic fluency and naming will produce scores that are equivalently impacted by both the aMCI and LLD. When comparing AD groups to LLD groups, AD has a significant incremental effect on semantic fluency and naming scores impairment, but not on phonemic fluency. In other words, in the identified studies, the semantic fluency or naming task significantly discriminated between AD and LLD scores but the phonemic fluency task did not.

The results on semantic fluency are coherent with a previous meta-analysis conducted by Henry and Crawford (2004), which showed that the adverse effect of AD on the performance of these tasks was greater than that of depression, although the meta-analysis included younger adults in the depressed groups, whereas our purpose was to assess the specificity of age in LLD.

We identified substantial heterogeneity between the studies included in the calculation of the pooled effect of LLD (when compared to the control group) for the results of the semantic, phonemic fluency and naming tasks. The linear regression model that explored the effect of age, level of depressive symptoms and level of education on these scores showed that level of education predicts the effect of depression on scores on these three tasks. To do this, we calculated ratios representing the magnitude of the difference in these variables between the LLD group and the control group for each study and introduced these ratios as variables in a linear regression to see which one best predicted the effect sizes of LLD on outcomes. Only the ratio of the difference in education level showed a significant effect in all three models.

This influence of education level on scores at semantic fluency was not true for MCI and AD groups. This may be explained by the fact that the overall effect of LLD is much lower and is more sensitive to secondary variables. It is worth noting that we also considered whether the different types of depression identified in the research could explain the substantial heterogeneity between their scores on these tasks. However, when tested by introducing this factor as a random variable in the linear regression, no statistical significance was found. The distribution of effect sizes across studies is not better explained by the variety of types of depression reported.

**Fig. 2:**
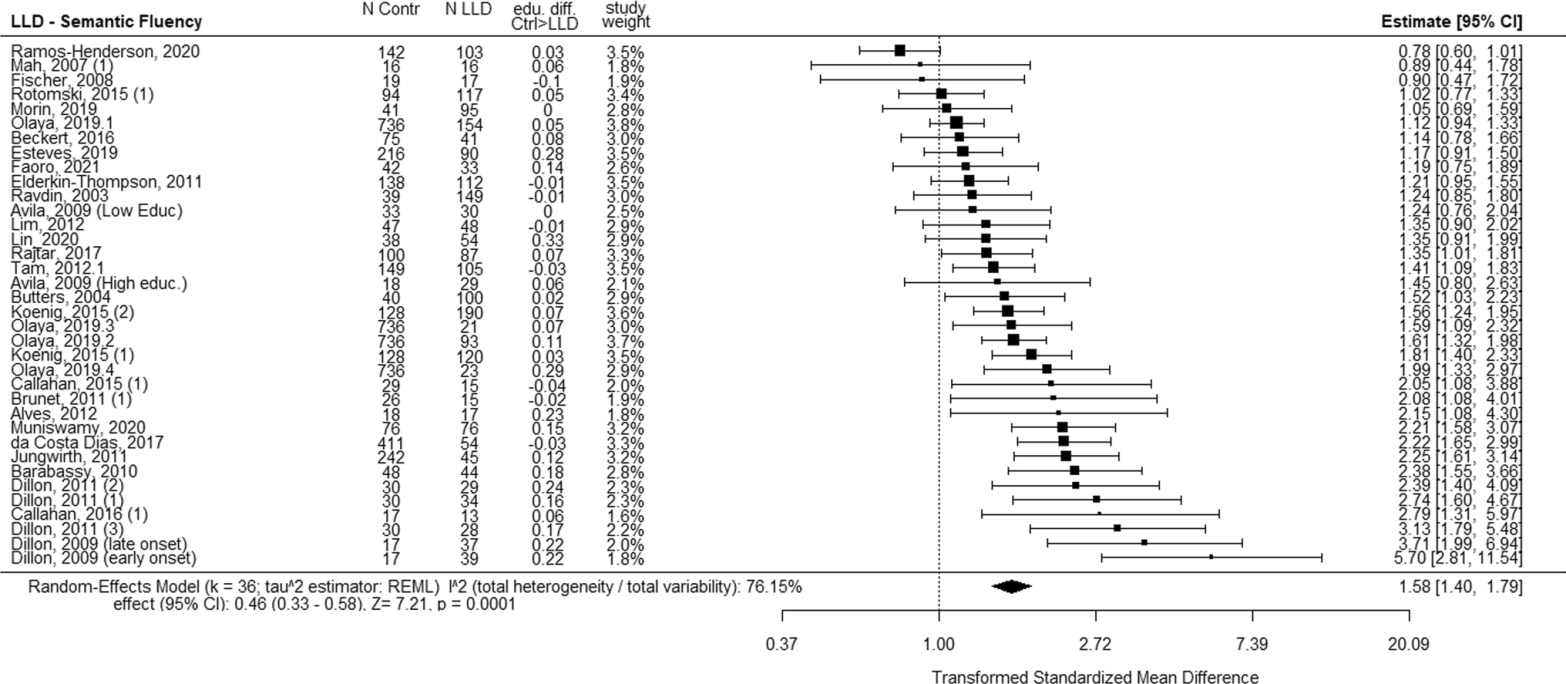
Forest plot of average effects of LLD (compared to the control group) for outcomes of semantic fluency, including differences in education. Note: when a study includes several LLD groups (see details in Table 1), each comparison corresponding to a LLD group is subject to an effect size. The "edu. diff. Ctrl > LLD" represents a ratio calculated to reflect the extent of the difference in educational level between the two groups being compared; positive values of this ratio indicate that the educational level of the control group was higher than that of the LLD group

**Fig. 3:**
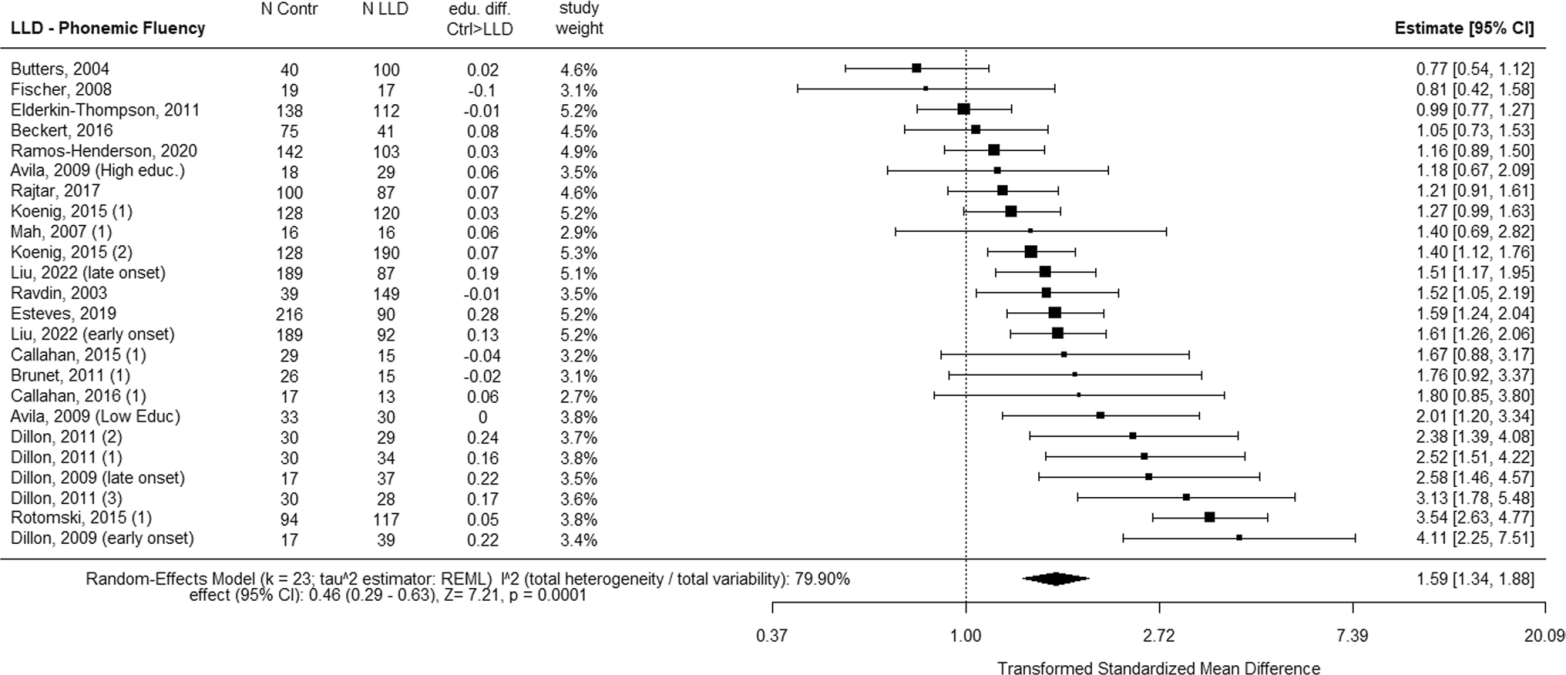
Forest plot of average effects of LLD (compared to the control group) for outcomes of phonemic fluency including differences in education. *Note:* Same as for Fig. 2

**Fig. 4:**
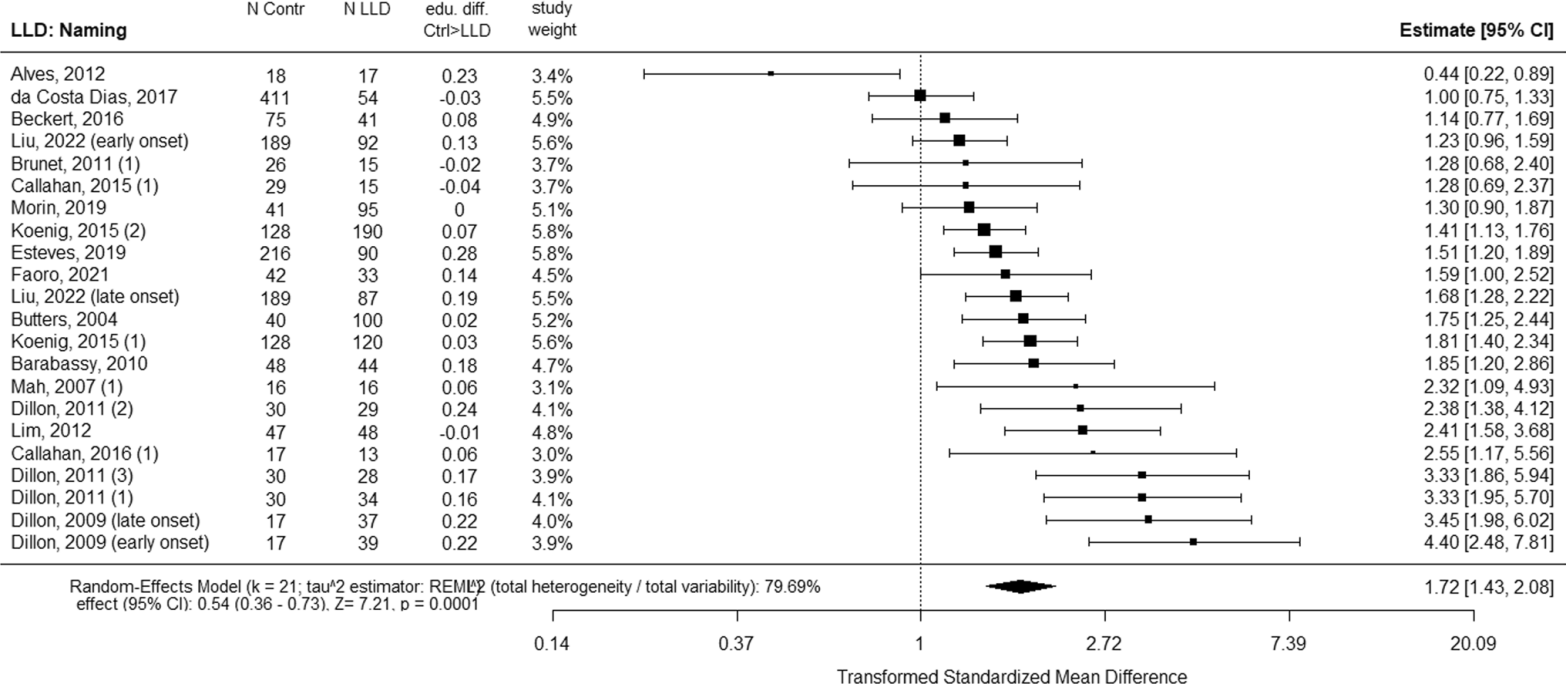
Forest plot of average effects of LLD (compared to the control group) for outcomes of Naming task including differences in education. *Note:* Same as for Fig. 2

However, despite the lack of proof by statistical significance, it is interesting to describe some methodological differences between the studies and to view them regarding the forest plot representing the distribution of LLD effects (compared to control groups) for the semantic fluency (Fig. 2), phonemic fluency (Fig. 3) and naming tasks (Fig. 4).

The studies that reported the most important effect of LLD on both fluency and naming are Dillon et al. (2009, 2011). The first study (Dillon et al. 2009), controlled the age at the first onset of depression between late- (> 60 years old) and early-onset (< 60 years old) depression (Dillon et al. 2009) was controlled. In the subsequent study (Dillon et al. 2011), they also controlled the variation between types of depression between groups with major depression, dysthymia, subsyndromal depression, and depression due to mild Alzheimer’s dementia (Dillon et al. 2011).

While in the studies by Dillon et al. all different groups of LLD had a major impairment on these three tasks, in Koenig et al. (2015), the distinction between two groups of depressed patients, differing in the severity of depression between euthymic and major depression, was not reflected in results of dramatically different magnitudes.

If we detail four other studies that examined the effect on semantic fluency, phonemic fluency and naming (Brunet et al. 2011; Butters et al. 2004; Callahan et al. 2015; Rajtar-Zembati et al. 2017), we see that Butters et al. (2004) showed an effect of LLD only on naming but did not report a control of the severity of the depressive status of the LLD group while they did control the age of onset of depression. Brunet et al. (2011) and Callahan et al. (2015) showed no significant effect of LLD on semantic fluency, phonemic fluency or confrontation naming. On a methodological level, Brunet et al. (2011) did control for the homogeneity of education and the level of depressive symptoms, but did not report information about the previous history of depression in the LLD groups. Callahan et al. (2015) strictly controlled the absence of neurocognitive disorder in the LLD group and the homogeneity of education between the groups. Rajtar-Zembati et al. (2017) that reported an effect on semantic fluency controlled that none of the participants had a previous history of depression, making it a late-onset LLD group, but reported that some of the individuals in LLD group were medicated with antidepressants (35%), and this variable was not controlled in the statistical analysis of the results (Rajtar-Zembaty et al. 2017).

Four studies showed an effect of LLD on scores for phonemic fluency but not for semantic fluency (Avila et al. 2009; Esteves et al. 2019; Ravdin et al. 2003; Rotomskis et al. 2015). Avila et al.’s (2009) research was strictly balanced for the similarity of education between groups. They also measured the severity of depressive symptoms in the depressed group, showing that depressive disorders were mostly mild-to-moderate. Rotomskis et al. (2015) did not provide the level of depressive symptoms for any of the groups or participants’ previous history of depression. However, the homogeneity of the education level was strictly controlled (Rotomskis et al. 2015). Esteves et al. (2019) reported an effect of LLD on phonemic fluency and on confrontation naming task, but not on semantic fluency. The article did not provide a diagnostic reference (neither DSM nor ICD) for the classification of LLD. Ravdin et al. (2003) strictly controlled the homogeneity of the education level between groups, but did not report the previous history of depression for the participants (Ravdin et al. 2003).

Four studies showed no effect of LLD on scores of either of the fluency task (Beckert et al. 2016; Elderkin-Thompson et al. 2011; Fischer et al. 2008; Mah et al. 2017). They reported strict control of non-dementia status for the participants of the LLD group. In Mah et al. (2017) and Fischer et al. (2008) all participants in the LLD group suffered from severe depression. In Elderkin-Thompson et al. (2011) the participants were separated into early- and late-onset LLD and drug free for antidepressant therapy at the time of the research (Elderkin-Thompson et al. 2011). In Beckert et al. (2016), the LLD group was of low education (half of the group had less than seven years of education and the other half had less than three years of education or were illiterate), the severity of depressive symptoms was not controlled as a variable, and participants were accepted in the research with Mini mental state evaluation (MMSE) scores of 22/30.

Five studies reported LLD scores only for semantic fluency and confrontation naming (Alves et al. 2012; Barabassy et al. 2010; da Costa Dias et al. 2017; Faoro et al. 2021; Morin et al. 2019). Barabassy et al. (2010) reported important differences in the level of education between groups, with a lower and more widely distributed level in the LLD group. In this study, the level of depressive symptoms was not reported in the control group (Barabassy et al. 2010). Da Costa Dias et al. (2017) compared groups of huge differences of sizes (411 controls for 54 LLD) (da Costa Dias et al. 2017). 

Liu et al. (2022) compared the early and late onset of LLD and balanced the level of the severity of depressive symptoms between them. The level of education was significantly lower for both depressive groups than for the control group. For the early-onset group, scores were significantly lower for phonemic fluency.

When it comes to the studies including also a comparison with a MCI or AD. Brunet et al. (2011) and Callahan et al. (2015) included two groups of aMCI, with and without depressive symptoms, and strictly controlled for the homogeneity of education between groups. Their results show that aMCI with depressive symptoms had significantly lower scores for semantic fluency and confrontation naming but not for phonemic fluency; aMCI without depressive symptoms had significantly lower scores only for semantic fluency but not for phonemic fluency or confrontation naming. Mah et al. (2017) included a group of patients with aMCI without depressive symptoms in their study. The groups were homogeneous with a high level of education (> 15 years of education). Their results showed no significant differences between groups for either fluencies or confrontation naming. Finally, all the studies including groups with AD (with or without depression) showed a significant effect of AD on the three tasks. In summary, the order of magnitude of the pathological situation effects on naming and both fluency was an overwhelming dominance of AD with a much weaker and more heterogeneous influence of LLD or aMCI, likely multi-determined. This leads us to conclude that measures of fluency and naming, while demonstrating high discriminatory value for AD, do not have the same clinical value for cognitive assessment to identify LLD and aMCI. However, the effect is still present and does not allow us to claim that in the case of LLD, these tasks must not be expected to be impaired, which denies the possibility of using them as discriminating variables in the differential diagnosis.

### Effects of LLD, aMCI AND AD ON low-executive tasks

Regarding tests in low-executive group, defined as not relying on a high-executive contribution, three studies (Brunet et al. 2011; Callahan et al. 2015, 2016) used the Pyramid and Palm Tree Test (PPTT) (Adlam et al. 2010). None of these studies showed an effect of LLD or aMCI. Notice that this one included groups of participants with a larger number of participants than in the other two studies (Brunet et al. 2011; Callahan et al. 2016). PPTT uses nonverbal responses to access semantic knowledge to identify that two images are semantically linked. It is a spotting task designed to reduce the reliance on other cognitive resources different from activation in the semantic representation system.

Two studies (Butters et al. 2004; Koenig et al. 2015) reported the results of a spot-the-word task (from the WAIS (Hartman 2009)) and did not show any significant effect of LLD on it. This task, also described as a lexical decision task, is conducted by presenting to the participant pairs of items comprising one word and one non-word, and requiring the subject to identify the word (Baddeley et al. 1993) and is reported to be a measure of cognitive ability that is resistant to the effects of brain injury because it relies on crystallized measures of verbal knowledge.

Two studies used the test of similarities (Fischer et al. 2008; Morin et al. 2019) to compare the LLD and control groups and showed no effect. In the similarities subtest, the participant must identify the similarities between two concepts, which requires the retrieval of knowledge of both concepts, but also abstract thinking skills and verbal reasoning. Three studies used a vocabulary subtest (Dillon et al. 2009; Faoro et al. 2021; Mah et al. 2017) that requires to define of up to 30 words. The results of the three studies differed: Dillon et al. (2009) and Faoro et al. (2021) show no significant effect of LLD, while Mah et al. (2017) found a significant effect of LLD on the performance of this task (and no effect of aMCI with depressive symptoms). Note that the vocabulary task of the WAIS includes several words highly linked to emotions (e.g., regrets, courage, remorse, etc.) that can have a negative effect on the compliance of depressed participants to complete the task.

Rotomskis et al. (2015) and Beckert et al. (2016) compared LLD and control groups using the language subtest of the ACE-R and did not report significant differences between LLD and control group but a significant effect of AD on the outcomes.

Finally, Elderkin-Thompson et al. (2006) compared major and minor LLD to a control group with a cognitive assessment including the learning, recall, and recognition tasks of the Californian Verbal and Learning Test (Elwood 1995). The semantic clustering strategy index was measured by the authors as an indicator of executive mediation for learning task performance. The results of the recall and recognition tasks did not significantly differ from one group to another, while the learning task did (*F* (8316) = 3.71; *p* < 0.001), as did the semantic clustering index (*F* (2160) = 4.79; *p* = 0.01). The authors interpreted this result as a demonstration that the relationship between depression and verbal learning tasks is mediated by executive ability, as quantified by semantic clustering. However, they did not show a distinct effect of minor or major depression on this indicator.

## General discussion

The cognitive complaints encountered in LLD make it difficult to distinguish between aMCI and AD based on an analysis of neurocognitive disorders. The hypothesis of the early impairment of semantic memory in AD and aMCI is considered a potential differential cognitive clue. By systematically seeking neuropsychological assessments of individuals with LLD, the present study included 31 studies representing 3291 controls and 2820 people with LLD. Wherever possible, studies that tested simultaneously groups with LLD, AD (or aMCI) were also included.

The meta-analysis of group comparisons of scores at tasks that were influenced by executive resources (verbal fluency or confrontation naming) showed a moderate effect of LLD on these tasks, which was less important than the effect of AD reported by previous meta-analyses (Henry and Crawford 2005; Laws et al. 2007, 2010). Then, the aMCI showed an incremental deleterious effect on semantic fluency, but not on phonemic fluency and naming. Finally, AD had an enhanced effect on semantic fluency and naming, but not on phonemic fluency. These results show that semantic fluency is sensitive to the difference in cognitive impairment encountered between LLD and aMCI (and AD), that naming only shows an incremental effect with AD and not with aMCI, and that phonemic fluency is not sensitive to the difference between LLD, aMCI or sometimes even AD. Research comparing aMCI with and without depressive symptoms shows that aMCI with depressive symptoms has the same pattern as AD (incremental deficits in semantic fluency and naming scores compared to LLD), but that in highly educated groups this pattern does not emerge, suggesting that compensatory mechanisms are at work. These results are coherent regard to the hypothesis that the presence of depressive symptoms during aMCI is linked to a major rate of conversation to AD (Invernizzi et al. 2021), and with the hypothesis of the specific impairment of the activation of the semantic representation system as one of the earliest signs of AD.

This result can provide complementary information about aMCI to that reported by Joubert et al. (2020) in a meta-analysis of the effect of MCI on semantic tests. Their results indicated a systematic effect of MCI on semantic tests when compared with healthy controls (Joubert et al. 2020). However, in their research, the selected semantic tests were confrontation naming, free recall of semantic information, and facilitated recall of semantic information. Confrontation naming and free recall of semantic information rely on self-initiated recall or retrieval of semantic information (such as verbal fluency).

In general, the outcomes from our pooled effect sizes support the idea that during the cognitive assessment of a person with LLD, verbal fluency scores are expected to be affected, but not to the same extent as in the case of AD. However, the difference in effect between LLD and aMCI is not as clear and does not allow fluency to be identified as a discriminating tool in a differential cognitive assessment between aMCI and LLD. Moreover, in the case of LLD, the results were strongly influenced by the heterogeneity of education levels between the groups, which makes it even more difficult to interpret the results. Our results show that differences in educational level between groups predict outcomes on the phonemic and semantic fluency and naming tasks, but do not predict outcomes of the other tasks. However, looking at each study separately, we could see that some studies had strictly controlled this balance of education between groups and sometimes showed effects and at other times not. The other possible sources of heterogeneity were the severity of the depression, the type of depression and the history of previous episodes. Future research on the cognitive profile in LLD should strictly control these three elements.

The hypothesis of late-onset LLD (i.e., without a history of depression earlier in life) reports that it is a frequent sign of a prodrome of AD (Heser et al. 2013; Ohanna et al. 2011), suggesting that when this is controlled for, the impact of this particular late-onset LLD would be more pronounced on semantic than phonemic fluency; however, this trend did not emerge. In the same way that some cognitive expressions specific to early AD do not materialize in aMCI, it is possible that this specific effect of AD on fluency is not so precocious as to make it an indication that certain types of late-onset LLD are to be observed as a possible prodrome of AD.

Since the included studies were not longitudinal, we can consider that the aMCI groups evaluated represents both those who evolve and those who remain stable; hence, we cannot draw any conclusions regarding the situation of aMCI intented to evolve into AD. An interesting line of research in this sense would be to conduct a meta-analysis on the effects of aMCI on these three tests, including only longitudinal research, allowing the classification of aMCI according to its evolution. At this point, however, our preliminary conclusion on the relationship between aMCI and verbal fluency and naming is that these tests are not sensitive enough to play a significant role in the cognitive profile of aMCI. Consistent with previous research (Henry and Crawford 2005; Laws et al. 2007, 2010), the incremental effect of AD is fully demonstrated for these tests, except for phonemic fluency.

One part of our review analyses the studies that have extracted results from experimental groups on tasks involving semantic retrieval, but assumed to require less, or at least less intense, executive input. These studies do not present fully unanimous results, but the purest semantic tasks (such as the PPTT), which are affected by AD, are spared in the case of LLD.

## Conclusion

The use of neuropsychological tests relying on semantic memory functioning but involving a strong executive component such as phonemic fluency, is not of differential diagnostic interest between LLD and AD (or aMCI) because, although of lesser magnitude, LLD in a large proportion of cases also has a negative impact on scores for these tasks. Moreover, there is considerable heterogeneity in the results of these tasks in LLD. In this sense, data such as the level of education, degree of severity of depression, its typology, and its history, must be strictly controlled in research linking this pathology to these tasks.

However, the use of semantic fluency, naming or of low-executive tests (that do not rely on the self-initiation of semantic information and therefore rely much less on executive functions) are significantly less affected by LLD than AD, which makes them relevant for differential diagnosis, in line with the hypothesis of a specific and early impairment of the activation of the semantic representation system in AD.

If a clear distinction exists between the absence of an effect of LLD and the significant effect of AD on the activation of the semantic representation system, further research must be conducted to specify the difference in impairment between these conditions on the executive semantics involved in self-initiated semantic activities. When the studies are not longitudinal, it is difficult to assert a link between the presence of depressive symptoms in aMCI as a sign that it is a prodromal aMCI to AD, confirmed by early semantic impairment. This hypothesis should also be explored through a meta-analysis of longitudinal studies on aMCI. However, our results show a clear discriminative character of these tasks in distinguishing between AD and LLD.

The exercise of pooling tests designed to assess semantic memory remains a difficult one in the framework of a systematic review of the literature. Indeed, the practice of using tests assessing semantic memory in a pure way (such as the camel and cactus test) remains rather confidential and we had to work with more disparate material, such as fluency or vocabulary subscales of intelligence tests. However, despite the heterogeneity encountered, our review shows that almost all the tests that rely on semantic memory but are also determined by other cognitive functions (such as executive functions for fluency or language for vocabulary tests) are affected by LLD. However, our results also show that for these tests, AD has a substantial incremental deleterious effect. Our results also show that tasks that minimized the use of any function other than activation and selection in semantic memory (typically semantic matching tasks, which are very sensitive to AD) show very normal results in the context of LLD. Finally, our results show that aMCI is a pathological condition whose cognitive impairment is similar to LLD, but that in the presence of aMCI and depressive symptoms simultaneously, the cognitive pattern becomes more similar to that of AD than LLD. Despite the limitations of this work, it provides an interesting basis for the differential analysis of the cognitive processes at work in these three pathologies and allows us to hypothesize, in particular, about the continuum that may link aMCI to AD, through the presence of depressive symptoms. Future work will have to quantify the difference in effect between aMCI and AD on these tests as a whole, to complete the analytical grid constructed around the neuropsychological assessment.

## Data Availability

https://osf.io/evrwf/?view_only=935fb2bcf5aa487f90ec22a034835b0a.

## Declarations

### General statement

We confirm that the manuscript has been read and approved by all named authors and that there are no other persons who satisfied the criteria for authorship but are not listed. We further confirm that the order of authors listed in the manuscript has been approved by all of us. We confirm that we have given due consideration to the protection of intellectual property associated with this work and that there are no impediments to publication, including the timing of publication, with respect to intellectual property. In so doing we confirm that we have followed the regulations of our institutions concerning intellectual property. We understand that the Corresponding Author is the sole contact for the Editorial process (including Editorial Manager and direct communications with the office). She is responsible for communicating with the other authors about progress, submissions of revisions and final approval of proofs. We confirm that we have provided a current, correct email address which is accessible by the Corresponding Author.
