# Correction: The role of semantic assessment in the differential diagnosis between late‑life depression and Alzheimer’s disease or amnestic mild cognitive impairment: systematic review and meta‑analysis

**DOI:** 10.1007/s10433-023-00783-w

**Published:** 2023-08-23

**Authors:** Sandra Invernizzi, Alice Bodart, Laurent Lefebvre, Isabelle Simoes Loureiro

**Affiliations:** 1 Department of Cognitive Psychology and Neuropsychology, University of Mons, Mons, Belgium; 2 Fonds National de La Recherche Scientifique, Brussells, Belgium

**Correction: European Journal of Ageing (2023) 20:34** 10.1007/s10433-023-00780-z

Following publication of the original article (Invernizzi 2023), the given and family names of [Sandra Invernizzi^1,2^ · Alice Bodart^1^ · Laurent Lefebvre^1^ · Isabelle Simoes Loureiro^1^] were incorrectly structured. The name were displayed correctly in all versions at the time of publication.

The original article has been corrected.
